# Family satisfaction with care provided to patients deceased in intensive care unit

**DOI:** 10.12669/pjms.40.10.8697

**Published:** 2024-11

**Authors:** Saleem Sharieff, Ayesha Sajjal, Asim Idrees, Wajid Ali Rafai

**Affiliations:** 1 Saleem Sharieff, MBBS, FCPS, FRCPC Senior Consultant Intensivist, Pakistan Kidney and Liver Institute and Research Center, Lahore - Pakistan; 2 Ayesha Sajjal, M.Phil Coordinator ICU, Pakistan Kidney and Liver Institute and Research Center, Lahore - Pakistan; 3 Asim Idrees, MBBS, FCPS. EDIC Consultant Intensivist, Pakistan Kidney and Liver Institute and Research Center, Lahore - Pakistan; 4 Wajid Ali Rafai, MBBS, MRCP Senior Registrar, Pakistan Kidney and Liver Institute and Research Center, Lahore - Pakistan

**Keywords:** *Intensive care unit*, Satisfaction, Caregiver

## Abstract

**Background & Objective:**

Patient’s family satisfaction is a crucial but often overlooked indicator of the quality of the intensive care unit (ICU). The goal was to assess how satisfied patients’ families were with the treatment they received for terminally ill patients, where we worked with them to create a care plan.

**Methods:**

The study was conducted in ICU at Pakistan Kidney and Liver Institute and Research Center (PKLI & RC), Lahore, Pakistan which is a quaternary care center between December 1^st^ 2022 and April 30^th^ 2023. We used the Family Satisfaction in the Intensive Care Unit 24 (FS-ICU 24) questionnaire to interview the next of kin of patients who passed away in our ICU.

**Results:**

Of the 330 patients admitted to the intensive care unit (ICU) between December 1, 2022, and April 30, 2023, 42 patients (12.7%) passed away. Mean age was 46.8 ± 14.5 years, with 28 men (66.7%) and 14 females (33.3%). Mean length of ICU stay was 5.57 ± 5.84 day. On a scale of 1-5 (1 = very Dissatisfied; 5 = fully satisfied) the overall family’s satisfaction score was 4.21 ± 0.52 with ICU consultants received a high satisfaction rating for informing families about their patients’ conditions and involving them in the decision-making process.

**Conclusion:**

The study showed involving family members in the care plan and helping them prepare for death not only helps them deal better but also makes them feel satisfied with the treatment they received in the intensive care unit, It makes them appreciate that they were able to participate in the decision-making process.

## INTRODUCTION

Patients and their families are anxious about the condition, procedures done in Intensive Care Unit (ICU), risk of complications, prognosis and other unfamiliar experiences due to critical illnesses. Many common complaint that are generally brought up by patients or their families includes lack of information provided during hospital stay, no one listen to them, lack of empathy, not engaging them in management plan, not explaining the prognosis and expected outcome. Also, families feel unsupportive emotionally and therefore unable to cope with poor outcome of their patient in difficult situation.

Poor satisfaction levels reflect a large difference between expectations and fulfilment of perceived needs, and may have implications for adherence to treatment that subsequently affects patient outcomes.^1^ Providing information about patient’s condition and outcome in the ICU to patients and families may help lowering anxiety, frustration, build up confidence, trust and satisfaction with healthcare team.

In order to improve the quality of care in ICU, it is important to get feedback from patients and their family members to address areas of concern. To our knowledge there is no such study reported from Pakistan that involved family satisfaction survey of deceased patients. The objective of the study was to assess how well families of patients who died in ICU were communicated with and to assess how our support and engagement of family in care plan help them to cope in this difficult situation. Therefore, this unique study provides information on how to improve quality of care and meet the expectations of patient’s families.

## METHODS

The survey consists of a questionnaire carried out in Intensive care unit (ICU) of Pakistan Kidney and Liver Institute and Research Centre (PKLI & RC), Lahore, Pakistan which is a quaternary care center, between December 1^st^ 2022 and April 30^th^ 2023. Our ICU has 14 surgical and eight medical beds and is a closed model under certified intensivists.

### Ethical approval:

Ethical approval has been obtained from the Institutional Review Board (PKLI-IRB/AP/134; dated July 20, 2023).

The Family Satisfaction in the ICU (FSICU) questionnaire is a patient family satisfaction questionnaire originally developed in 2003 by a group of health care professionals in Canada: the Canadian Researchers at the End of Life Network (CARENET).^2^ The questionnaire has been validated for use in North America and Europe,^3^-^6^ and has been adopted by other centers internationally. It consists of open-ended questions on various items focusing on satisfaction with ICU care and family’s involvement in decision making process of critically ill patients. During the study period a total of 330 patients were admitted out of which 42 died (12.7%). Using this standardized questionnaire (Appendix-1) we interviewed families of patients who passed away in ICU.

**Appendix-1:** Questionnaire for PKLI Family satisfaction Survey in Intensive Care Unit (PKLI FSS-ICU).

***Family satisfaction Survey (FSS)***	
***How did we treat your family member (the patient)?***	
Q1	Concern and caring by ICU staff? The courtesy, respect and compassion your family member (the patient) was given.
Q2	Symptom management? How well the ICU staff assessed and treated your family. member’s symptoms - Pain
Q3	Symptom management? How well the ICU staff assessed and treated your family. member’s symptoms – Breathlessness
Q4	Symptom management? How well the ICU staff assessed and treated your family. member’s symptoms – Agitation
Q5	Symptom management? How well the ICU staff assessed and treated your family. member’s symptoms – Anxiety
Q6	Symptom management? How well the ICU staff assessed and treated your family. member’s symptoms – Depression
***How did we treat you?***	
Q7	Consideration of your needs? How well the ICU staff showed an interest in your needs.
Q8	Emotional support? How well the ICU staff provided emotional support.
Q9	Co-ordination of care? The teamwork of all the ICU staff who took care of your family member.
Q10	Concern and caring by ICU staff? The courtesy, respect and compassion you were given. Anxiety? How well supported you felt by the ICU staff in relieving anxiety.
Q11	Depression? How well supported you felt by the ICU staff in relieving depression.
Q12	Anxiety? How well supported you felt by the ICU staff in relieving anxiety.
***Nurses***	
Q13	Skill and competence of ICU nurses? How well the nurses cared for your family member.
Q14	Frequency of communication with ICU nurses? How often the nurses communicated to you about your family member’s condition.
***Doctors***	
Q15	Skill and competence of ICU doctors? How well doctors cared for your family member.
***The ICU***	
Q16	Are you satisfied from ICU doctors including consultants?
***Level/amount of health care***	
Q17	How satisfied were you with the level or amount of health care your family member received in the ICU?
***Family satisfaction with information (FSI)***	
Q18	Understanding and Satisfaction with information provided by the ICU doctors, nurses?
***Family satisfaction with decision-making process (FSDM)***	
Q19	Did you feel supported/included during the decision-making process?
Q20	Overall satisfaction from care provided in ICU

In this cross-sectional study we measured the satisfaction levels of patient’s family member with ICU care and in decision-making process. Independent factors that can affect satisfaction were divided into four sections and different questions were asked from families in each section of questionnaire to gather more and more information on care provided at different levels. The overall satisfaction with the composite score or provisional structure derived from items in this study is not easily measured. Consequently, we were able to assess each question in detail and ask for the family member’s overall experience at the end of the survey. The scoring was done on a scale of 1-5 with score of 1 = Very Dissatisfied; 2 = Slightly Dissatisfied; 3 = Mostly Satisfied; 4 = Very Satisfied and 5 = Completely Satisfied.

### Inclusion criteria:

Adult family member who should be the next of kin and caregiver for the patient requiring ICU admission. Pediatric patients were excluded from the study. Demographic information like gender, age and relationship with the patient were collected followed by ratings on satisfaction from patient’s relatives focusing on care, communication and support provided to them during the ICU stay of their patient. The questionnaire was filled in by our coordinator, who was not directly involved in patient care, after the death of patient and gathered information constituting entire ICU experience.

## RESULTS

Out of total of 330 admissions in Intensive care unit (ICU), 288 (87.3%) survived while 42 patients (12.7%) who passed away were enrolled in the study. Demographic information as well as overall satisfaction score, is shown in Table-I. Among 42 deceased patients there were 28 males (66.7%) and 14 females (33.3%) and mean age was 46.8 ± 14.5 years. Mean length of ICU stay was 5.57 ± 5.84. On a scale of 1-5 (one was Very Dissatisfied while five was fully satisfied from services provided in ICU) the family’s satisfaction score of deceased patients was 4.21±0.52. The table also shows the breakdown of 330 cases and deaths in each category. Mortality was highest among medical cases given the fact that those patients already had underlying end-stage liver and kidney disease from various causes. The most common cause of death was septic shock and multi-organ failure.

**Table-I:** Demographics information of patients and next of Kin.

Variables	Values
Mean Age of Patients (Years)	46.86 ± 14.55
Mean Length of ICU stay (Days)	5.57 ± 5.84
Patient’s Gender	Male: 28 (66.7%) Females: 14 (33.3%)
Mean Age of Patient’s next of kin (Years)	37.86 ± 6.04
Next of Kin	Spouse: 6 (14.3%)
	Sibling: 14 (33.3%)
	Children: 15 (35.7%)
	Parent:4 (9.5%)
	Other Relative: 3 (7.2%)
Gender of next of Kin	Male: 27 (64.3%) Females: 15 (35.7%)
Liver Transplant recipient (Total = 41)	Expired = 4 (9.76%)
Liver Donor (Total = 41)	Expired = 0
Kidney Transplant Recipient (Total = 33)	Expired = 1 (3.03%)
Kidney Donor (Total = 33)	Expired = 0
Other Surgeries (Total = 119)	Expired = 3 (2.52%)
Medical admissions (Total = 63)	Expired = 34 (53.97%)
Deceased patients’ family’s satisfaction score according to diagnosis:	
Liver transplant	4.25± 0.96
Kidney transplant	4.00
Other surgeries	4.33 ±0.58
Medical cases	4.23 ± 0.49
Overall patients’ family’s satisfaction score (On a scale of 1-5; where 1 is very dissatisfied and 5 is completely satisfied).	4.21 ± 0.52

In answer to our questionnaire, the patient’s family gave us an overall satisfaction score of 4.21±0.52, the highest score given by family was on their satisfaction towards ICU consultants followed by information provided to them during ICU stay, their involvement in decision making and courtesy and respect given to them by ICU staff. The lowest scores were for support by ICU staff during anxiety followed by patient’s symptoms management especially pain, aggressiveness and delirium, (Table-II).

**Table-II:** Satisfaction survey from families of expired patients. (N=42).

Variables	Very Dissatisfied	Slightly Dissatisfied	Mostly Satisfied	Very Satisfied	Completely satisfied	Mean (±SD)
***A: How did we treat your patient?***						
Concern and caring by ICU staff	0	0	4 (9.5%)	25 (59.5%)	13 (31%)	4.21±0.61
Symptoms management of your patient	0	0	3 (7.1 %)	34 (81%)	5 (11.9%)	4.05±0.44
***B: How much do we support you?***						
How satisfy are you with treatment is ICU	0	0	3 (7.1%)	27 (64.3%)	12 (28.6%)	4.21±0.56
Emotional support provided to family by ICU staff	0	0	5 (11.9%)	25 (59.5%)	12 (28.6%)	4.17±0.62
Co-ordination of care with family	0	0	5 (11.9%)	25 (59.5%)	12 (28.6%)	4.17±0.62
Concern and caring by ICU staff like courtesy and respect	0	0	3 (7.1%)	22 (52.4%)	17 (40.5%)	4.33±0.61
support by ICU staff during depression	0	0	5 (11.9%)	26 (61.9%)	11 (26.2%)	4.14± 0.61
support by ICU staff during Anxiety	0	0	6 (14.3%)	29 (69%)	7 (16.7%)	4.02 ± 0.56
***C: How satisfied are you with ICU staff?***						
Skill and competence of ICU nurse	0	0	4 (9.5%)	22 (52.4%)	16 (38.1%)	4.23 ± 0.64
Frequency and communication with family by ICU nurse	0	0	2 (4.8%)	28 (66.7%)	12 (28.6%)	4.24±0.53
Skill and competence of ICU Consultant	0	0	3 (7.1%)	26 (61.9%)	13 (31%)	4.24± 0.58
Are you satisfied from ICU Consultant	0	0	1 (2.1%)	21 (50%)	20 (47.6%)	4.45± 0.55
Are you satisfied from ICU care	0	0	2 (4.8%)	28 (66.7%)	12 (28.6%)	4.23±0.53
***D: How was overall ICU care and your experience in ICU?***						
Information provided to family	0	0	1 (2.1%)	22 (52.4%)	19 (45.2%)	4.43±0.55
support provided during decision making	0	0	0	28 (66.7%)	14 (33.3%)	4.33±0.48
Overall satisfaction of family	0	0	2 (4.8%)	29 (69%)	11 (26.2%)	4.21±0.52

## DISCUSSION

This study indicates that effective communication and participation in decision making processes can be a source of satisfaction for families. The importance of empathy and family involvement in decision making, with a view to measuring satisfaction at all levels, was emphasized in this study. Family members’ needs and wishes are important in terms of both their role as supporters for the patients, and their own personal needs. This is the first ever satisfaction survey from families of deceased patients in ICU reported from Pakistan that provides information about deceased patients’ families’ experience with ICU quality of care.

PKLI is the largest transplant center of Pakistan and our patient population mostly include end-stage liver and kidney disease patients. Mortality rate in our ICU was 12.7% which is much less than reported by Divatia et al.^7^ (18.1%) from India, Acharya et al.^8^ (32.8%) from Nepal and Feroz SH et al.^9^ (22.66%). The later reported decrease in mortality from 1st year (26.11%) to 2nd year (18.5%) in his 2-year study due to improvement in their ICU care. Intensive care units generally have high mortality rate and services including patient demographic features, natural history of diseases, management, technology used, and infrastructure are all linked together. The high mortality can be attributed to the severity of illness on admission including the complex nature of patients the hospital receives being a quaternary referral and largest transplant center of the country. Better intensive care management at administrative and academic levels can result in significant reduction in overall mortality of ICU.^10^

Clinical audit is a valuable part of developed countries and is used to identify and improve the deficiencies in daily practice, unlike developing countries.^11^ A historical review of audits shows that the objective and role of auditors are constantly evolving, and can improve the evaluation of health care management’s structure, process, and outcome. It will help enhance the system to work with standard to develop good clinical practice and beneficial for patients and clinicians.

Dodek et al.^12^ described quality enhancement as a key goal when charting family satisfaction. Although there are several different general measures of satisfaction exists in literature but typically, both general and specific measures of satisfaction focus on areas such as provision of information, empathy with the patient, and attitude to the patient, access to and continuity with the caregiver and technical competence. Also important are the access to information about patient’s care and the quality of interpersonal relationships as key elements of patient and their family’s satisfaction. Therefore, measures of satisfaction should be specific, differentiated and when possible, multidimensional.

In general, these concerns include the management of symptoms in patients, information for family members, emotional support to families, consistency of information and inclusion into decision making processes. Some families mentioned the need for support from health-care team and when offered counselling they felt much comfortable in coping with the situation.

A series of studies have highlighted the importance of communication^5^,^13^-^18^ and skills of health care professionals and their behavior when dealing with patients and families have been largely affected by the perception of physician competence and a high degree of concern shown to patients and family members. Anxiety among family members of patients in the Intensive Care Unit has been reduced by a longer period of communication between health care teams and families.^17^ In our study, the highest satisfaction score was towards ICU consultants followed by family members’ ease of getting information, involvement in decision making and courtesy and respect given to them by ICU team.

Studies have shown that the significance of “VALUE” helps (evaluating and valuing what family members say, acknowledging family members’ feelings, listening, asking open-ended questions, and eliciting questions from family members) aids in addressing symptoms of anxiety, depression, and post-traumatic stress disorder.^18^ Family satisfaction in Intensive Care Units has also increased through education and training of communication skills, particularly the ability to listen with a view to giving families more opportunities for speaking at meetings.^19^

Many families experience the time spent in the ICU as challenging and full of uncertainty regarding the intensive care patient’s condition, treatment and prognosis. They felt frustrated, emotional, tearful and frightened when they first come to ICU and sometimes depressed at different stages of patient’s condition and didn’t always know why. The thought that their loved one is critical or nearly died, had a huge impact on most people and everyone dealt with this in different ways. When they realized how much love and support there was around them, some of them felt emotional as well.

While in ICU there is always some element of aggressiveness towards treatment from family members as they wish to receive all possible treatment and to get more and more involved in participation during decision-making process. Some people felt paranoid and become aggressive during the terminal stage of their patient. These factors have led to the ICU being in a difficult position and they are only solving problems by involving them in decision making processes and good communication. Therefore, our survey also tried to determine the extent to which patient families were satisfied with the aggressiveness of the care received, and the extent to which they had been involved in decision-making around aggressiveness of care thereby setting up the goals of care. Studies show that there may be a high prevalence among family members of depression and post-traumatic stress disorder (PTSD) at the end of an ICU stay.^20^,^21^ Families seek more support to relief their anxiety and thought that their patient’s symptoms especially pain, aggressiveness and delirium could be better control.

Unfortunately audit on medical practice is not routinely done in Pakistan and there is hardly any data available. Most of the audit are on mortality or on demographics of patient population, and, in our search, we could not find any audit or survey from Pakistan comprising patient’s family satisfaction from health care provided to the deceased patients. Therefore, it’s important to know the patient’s family perspective on health care services provide to their patient who unfortunately could not survive despite all efforts and management offered in ICU to improve the quality and standard of care.

We found that the proper communication with families and involving them in decision making process led to better satisfaction from care provided as well as minimizes the trauma that family experiences at the time of death of their loved ones. This is a unique experience from a large ICU in Pakistan which shows a high satisfaction scores in most items; however, there is always room for further improvement specially focusing towards management of patients’ symptoms like agitation, emotional support for the family, consistency of information and inclusion in and support during decision-making processes.

### Limitations:

Major study’s limitations is that, being a quaternary care hospital, our hospital specializes in treating renal and liver disorders. As a result, it was unable to generalize the findings. Also, we were unable to account for variations in the diagnostic standards, the severity of the initial illness, or the other treatment measures used.

## CONCLUSION

Patient and their family satisfaction are an important measure of ICU quality that demonstrate accountability and enhance patient care. Family members play a key role as both mediators of the intensive care patient’s needs and wishes and as a health-promoting resource that can improve patient outcomes. Our study showed good communication skills and involvement of families of patients in decision making process leads to family’s satisfaction even in patients who could not survive from their critical illness.

### Authors` Contribution:

**SS:** Principal investigator, study design, central idea, analysis, literature search and writing of manuscript. **AS, AI, WAR:** Data collection and entry, analysis, literature search and Review. All authors have read the manuscript and are responsible for the accuracy of the study.
